# Randomized Controlled Trial of Clozapine and CBT for First-Episode Psychosis with Enduring Positive Symptoms: A Pilot Study

**DOI:** 10.1155/2011/394896

**Published:** 2011-03-30

**Authors:** J. Edwards, J. Cocks, P. Burnett, D. Maud, L. Wong, H. P. Yuen, S. M. Harrigan, T. Herrman-Doig, B. Murphy, D. Wade, P. D. McGorry

**Affiliations:** OrygenYouth Health Centre for Youth Mental Health, University of Melbourne, Locked Bag 10, Parkville, VIC 3052, Australia

## Abstract

Here we report the results of a pilot study investigating the relative and combined effects of a 12 week course of clozapine and CBT in first-episode psychosis patients with prominent ongoing positive symptoms following their initial treatment. Patients from our early psychosis service who met the inclusion criteria (*n* = 48) were randomized to one of four treatment groups: clozapine, clozapine plus CBT, thioridazine, or thioridazine plus CBT. The degree of psychopathology and functionality of all participants was measured at baseline then again at 6, 12 and 24 weeks, and the treatment outcomes for each group determined by statistical analysis. A substantial proportion (52%) of those treated with clozapine achieved symptomatic remission, as compared to 35% of those who were treated with thioridazine. Overall, those who received clozapine responded more rapidly to treatment than those receiving the alternative treatments. Interestingly, during the early treatment phase CBT appeared to reduce the intensity of both positive and negative symptoms and thus the time taken to respond to treatment, as well having as a stabilizing effect over time.

## 1. Introduction

At least 10% of individuals experiencing a first episode of psychosis do not fully remit in the 12 months following the initiation of treatment [1, 2]. Given that prominent positive symptoms at 6-month followup predicts poor functioning [3], optimal treatments at this phase of illness are required to maximize the possibility of recovery. Earlier initiation of clozapine treatment has been proposed by the American Psychiatric Association [4] and the Royal Australian and New Zealand College of Psychiatry [5]. Outcome studies of efficacy in earlier phases of the illness indicate both better symptomatic and functional improvement in earlier stages of the illness [6], outcomes which can also be enhanced with psychological treatments such as cognitive behavioural therapy (CBT). 

CBT has emerged as one of the most effective adjunctive psychological treatments for treating positive symptoms during a first episode of psychosis [7–10]. Despite this increasing focus on psychological and pharmacological treatments in early intervention, there have been no studies evaluating the use of CBT in combination with clozapine in the subgroup of patients showing signs of early treatment resistance in first episode psychosis (FEP, i.e., prior to the emergence of sustained or established treatment resistance). This study was designed as a pilot investigation of the relative and combined efficacy of CBT and clozapine for reducing persistent positive symptoms following the initial treatment phase of first episode psychosis. We hypothesized that clozapine treatment would give a greater reduction in positive symptoms compared to a standard antipsychotic (thioridazine), that CBT would produce better outcomes compared to treatment without adjunctive CBT, and that the combination of clozapine and CBT would have a cumulative effect in heightening positive results from either therapy alone. 

Our data show that CBT appears to have a beneficial effect, particularly in the early phases of treatment, by reducing the intensity of both positive and negative symptoms and thus the time taken to respond to treatment. Interestingly, we found that the combination of CBT and thioridazine was equally as effective as clozapine alone, suggesting that CBT may well provide another tool for the management of persistent symptoms in FEP patients.

## 2. Methods

### 2.1. Participants

Participants were sourced from consecutive admissions to the Early Psychosis Prevention and Intervention Centre (EPPIC) at the Orygen Youth Health Centre for Youth Mental Health in Melbourne, Australia. The EPPIC program provides a comprehensive, integrated, community-based treatment program to FEP patients aged 15–29 years residing in the service catchment area [11]. All FEP patients entering EPPIC are screened for ongoing positive and negative symptoms at 9 and 12 weeks after the initiation of antipsychotic treatment, and those with ongoing positive symptoms at 12 weeks are managed by the TREAT team (treatment resistance early assessment team) in conjunction with their psychiatrist and case manager [12]. The TREAT team focuses on an assessment of the patient, including a physical assessment, and the nature and effectiveness of the pharmacological, psychological, and social interventions applied over the previous 12 weeks. Treatment principles include the active pursuit of an initially low-dose antipsychotic medication strategy, an expectation of at least two adequate trials (each a minimum of 6 weeks in duration) of an atypical antipsychotic within a 3-month period, the early introduction of clozapine, and intensive psychosocial therapy. The inclusion criteria for the study were: experiencing a first treated episode of a psychotic disorder that fulfilled the DSM-IV criteria for a diagnosis of schizophrenia, schizophreniform disorder, delusional disorder, or psychotic disorder not otherwise specified; being registered with EPPIC for 12 to 26 weeks; and continuing to experience moderate to severe positive symptoms, defined as a score ≥4 on at least one of the hallucinations, unusual thought content, and conceptual disorganisation items of the expanded version of the brief psychiatric rating scale (BPRS; [13]), with a score of not less than 3 on these items for a period of 14 consecutive days or more during the preceding 12 weeks. All participants had been treated with at least one atypical antipsychotic (usually risperidone, olanzapine or quetiapine) at doses up to 500 mg chlorpromazine equivalence (if tolerated), with demonstrated medication compliance for at least the past 4 weeks. Exclusion criteria were an organic mental disorder, pregnancy or lactation, requiring antidepressant medication, a mood stabiliser or ECT, and a history of drug-induced granulocytopenia.

### 2.2. Study Design and Interventions

**Figure 1:**
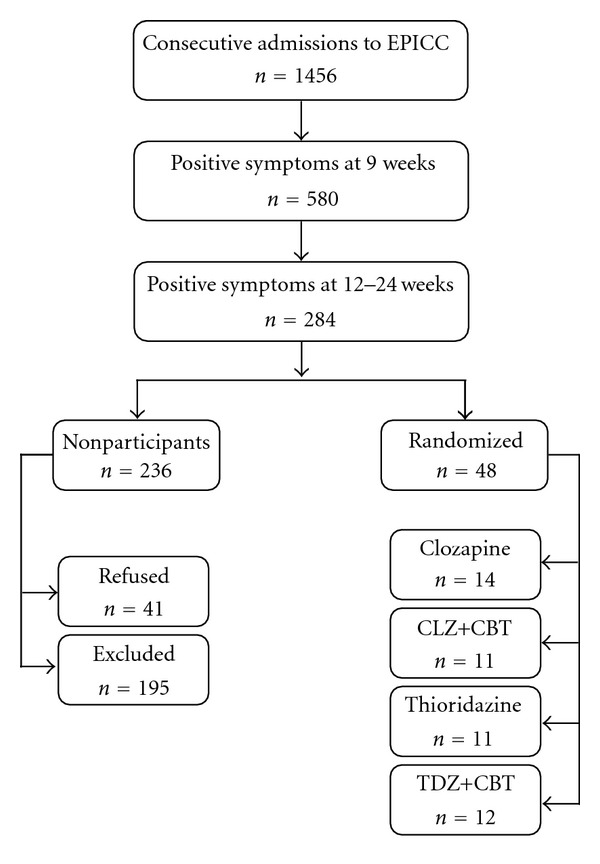
Participant flow chart.

This study was conducted as a single-blind randomized controlled trial, with a 12-week treatment phase and a 12-week followup phase, and was fully approved by the North-Western Mental Health Ethics Committee. Recruitment began in September 1996 and ended in March 2002, and of the 89 patients who met the eligibility criteria, 48 consented to participate in the study (Figure 1). These were randomized into four groups: clozapine (CLZ), CLZ+CBT, thioridazine (TDZ), and TDZ+CBT. Thioridazine was selected as the control antipsychotic due to its side-effect profile, which is similar to that of clozapine with regard to sedation, anticholinergic actions, and extrapyramidal side effects. Thioridazine and clozapine were considered dose equivalent. Participants commenced treatment on at a dose of 12.5 mg/day which was titrated upwards in 25 mg/day increments up to a maximum dose of 300 mg/day, depending on clinical response. During Week 4 of the study, patients who still showed an inadequate response had their medication increased to a maximum of 400 mg/day, which could be further increased to a maximum of 600 mg/daily if necessary during Week 5, after which all patients were maintained on their current medication dose. Additional pharmacological interventions (e.g., benzodiazepines) were provided as clinically indicated. Individuals receiving clozapine were monitored for haematological and cardiac complications according to the manufacturer's protocol and were withdrawn if the total white cell count fell below 3.0 × 10/L or the neutrophil count fell below 1.5 × 10/L.

A manualized CBT program, the systematic treatment of persistent psychosis (STOPP, [14]) was devised to target enduring positive symptoms and related patient needs. The therapy was conducted twice weekly for 12 weeks, with a minimum attendance of 15 sessions required. In addition, all participants received routine clinical care, which included access to a 24-hour mobile assessment and treatment team, inpatient service, case management, and psychiatric review. Patients were seen weekly by a psychiatrist/psychiatry registrar for the duration of the trial, and all participants not receiving CBT attended weekly case management sessions. Given the sensitivity of this patient group, where possible all participants remained with a single case manager, and all assessments were made as part of routine clinical care within the EPPIC program.

### 2.3. Measures

Mental state was determined with the expanded version of the BPRS [13]. The BPRS psychotic symptoms subscale (BPRS-P) was used to assess positive symptoms, while the scale for the assessment of negative symptoms (SANS; [15]) and the short form of the Beck depression inventory (BDI; [16]) assessed levels of negative symptoms and depression, respectively. The clinical global impression (CGI; [17]) was used to measure the severity of psychotic disorder, as well as the degree of improvement since baseline. Psychosocial functioning was assessed using the social and occupational functioning assessment scale (SOFAS; [18]) and quality of life survey (QLS; [19]). 

Demographic information, medication history and the duration of untreated psychosis (DUP) were collected at the baseline interview, and all participants were assessed using the royal park multidiagnostic instrument for psychosis (RPMIP; [20, 21]) or the SCID [22], as well as the BPRS, SANS, BDI, SOFAS, and QLS. The BPRS, SANS, and CGI were repeated fortnightly for the 12 weeks of the trial phase, and then monthly until trial completion. Information regarding the number of outpatient contact hours, the number of days of 24-hour home treatment and/or inpatient care, and weekly medication (maximum dose) were recorded at baseline and over the 12 weeks of the trial phase, and at trial completion, by retrospective examination of file notes and discussion with the case manager. All diagnoses were reviewed at 12 weeks by two senior clinicians, and in all cases diagnoses were confirmed as stable.

### 2.4. Statistical Analysis

The data was analysed according to the intention-to-treat (ITT) principle. Missing data was handled by using multiple imputation undertaken with S-PLUS 6.1 software. The effects of medication and CBT were investigated for each outcome measure in terms of the change in score from baseline to 6, 12, and 24 weeks. ANCOVA models were employed: the dependent variable was the change in score, and the independent variables included the baseline score, gender, and log transformed DUP as covariates, a medication factor (CLZ versus TDZ), a CBT factor (CBT versus no CBT), and the interaction between these factors. Complete-case analysis (i.e., using only those cases with nonmissing values) was performed as a complementary strategy. Three patients were recorded as experiencing problems with medication compliance, and thus a second ITT analysis was performed after excluding these patients from the data set, as well as a second complete-case analysis. 

All statistical tests were two-tailed, and results were regarded as significant at or below the 5% probability level. Correlation effect sizes were examined (*r* = 0.10, small; *r* = 0.3, moderate; and *r* = 0.5, large effect). Linear mixed effects modelling was used to compare the extent of improvement on outcome measures across time, and survival analysis was used to compare the different treatments in terms of time to first remission. The primary outcome measure was symptomatic remission, which was defined as a score of “mild” or less on each of the three items of the BPRS-P and a CGI severity item rating of “mild” or less. Secondary outcome measures were psychiatric symptoms, psychosocial functioning, and quality of life, which were assessed using the BPRS, SANS, BDI, CGI, SOFAS, and QLS scales.

## 3. Results

### 3.1. Participants

The participant flow chart is contained in Figure 1. The number of patients screened over the 5.5 years of the study recruitment period is consistent with the average EPPIC intake of 260 cases per annum [23]. The proportion of individuals with enduring positive symptoms at 12 weeks was examined on a yearly basis and showed a stable pattern with demographics similar to the total EPPIC cohort [23]. A total of 236 patients with persistent positive symptoms did not participate in the trial. Of these, 195 were considered ineligible for reasons such as inadequate medication dose (23.4%), being involved with EPPIC for longer than 24 weeks (10.2%), and resolution of positive symptoms (28.6%), while a further 41 eligible patients refused to participate. Individuals who agreed to randomization (*n* = 48) were comparable to those who refused randomization (*n* = 41) in gender (*P* = .61) and age (participants: mean = 21.4, SD = 3.5; refusers: mean = 21.0, SD = 3.3, *P* = .56).  Table 1 outlines the demographic and intervention data for the four treatment groups.

### 3.2. Outcome Measures

Only the results from the ITT analysis including the data from all 48 participants is presented here, since a complementary complete-case analysis excluding the data from the three participants considered noncompliant with their medication did not significantly change any of the outcomes.  Table 2 shows the data for the mean symptom measures for each group at baseline, week 12, and week 24. While groups varied on individual measures, there was no evidence of a systematic difference in functioning at baseline between the treatment groups.

At 12 weeks, all four groups showed improvements for each of the psychopathological outcomes measured. As expected, clozapine was more effective at reducing the level of positive symptoms than thioridazine over this timeframe, but interestingly, the addition of CBT gave a similar degree of improvement in positive symptoms to that seen with clozapine treatment alone (Table 2). The most notable improvement was seen for the SANS, where the both the CLZ and TDZ groups showed reductions to 83% and 86% of their mean baseline scores, respectively, in line with the general improvements seen across all the outcome measures. However, combining CBT with either medication resulted in a larger reduction in the SANS scores for both groups, to approximately 65% of their mean baseline scores (Table 2). These improvements were statistically significant (*P* = .049), with a moderate effect size of 0.41. The other statistically significant improvement seen at this time was for the BDI, where the CLZ group showed a 68% reduction relative to its mean baseline score (*P* = .044, effect size 0.4), while the other three treatment groups showed reductions to 80%–95% of their baseline values, again in line with the general levels of improvement seen for all measures. With regard to overall functioning, the CGI, SOFAS, and QLS scales all revealed modest general improvements for all groups, with no significant differences between the treatment groups.

The overall general improvement seen at 12 weeks was maintained at 24 weeks, though further improvement was seen for certain measures. With regard to positive symptoms, the TDZ and CLZ groups both maintained the initial improvements seen at 12 weeks. Significantly, the combination of medication and CBT resulted in a further moderate improvement in both treatment groups on all symptomatic measures, with both groups showing a greater improvement than that seen after treatment with medication alone. This effect approached statistical significance (*P* = .06) for the BPRS-P, where the CLZ+CBT and TDZ+CBT groups showed reductions to 65% and 58% of their mean baseline values, respectively. In terms of negative symptoms, the CLZ and TDZ groups both showed further improvement, with SANS scores of approximately 78% and 71% of their mean baseline values, while the CLZ+CBT and TDZ+CBT groups maintained similar levels of improvement to those seen at 12 weeks. The differences in score between the medication alone and medication + CBT groups seen at 12 weeks suggests an early beneficial effect for CBT on negative symptoms, which became less apparent by 24 weeks, since no significant difference was found between the treatment groups at this time. With respect to depressive symptoms, the TDZ and CLZ groups maintained their improvements seen at 12 weeks, while the TDZ+CBT group showed a statistically significant improvement, with a 41% reduction relative to the mean baseline score apparent (*P* = .002, effect size 0.53). Paradoxically, this was not seen in the CLZ+CBT group, whose mean BDI score remained stable with a 10% improvement over the mean baseline score, as opposed to the 30% improvement seen with CLZ treatment alone. Once again, the functional outcome measures remained stable, again showing a modest improvement over their baseline levels.

### 3.3. Remission Status and Time to First Remission

The remission status of each participant was recorded at each assessment visit, with symptomatic remission being defined as a score of 3 or less on each item of the BPRS positive subscale (unusual thought content, hallucinations, and conceptual disorganization) and a CGI severity rating of mild or less. By the end of the 24-week study period, 4 of the 11 participants (36.4%) in the TDZ group had attained remission at least once, while 4 of the 12 participants (33.3%) in the TDZ+CBT group attained remission. In the CLZ group, 7 of the 14 participants attained remission, while 6 of the 11 participants (54.5%) of the CLZ+CBT group remitted at some point during the study. 

**Figure 2:**
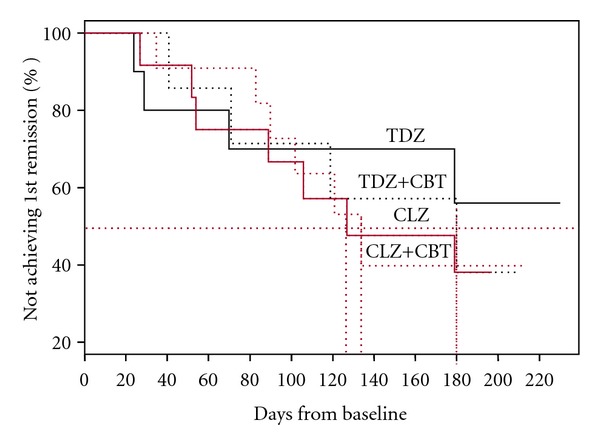
Survival analysis for time to first remission.

Survival analysis for the time to first remission was performed for each group using the remission status of each subject and the corresponding time measurements (i.e., time to first remission or time remaining unremitted), and is shown in Figure 2. Overall, 21 of the 48 participants, or 43.8%, remitted at some time during the 24-week study period, indicating that with appropriate treatment, symptomatic remission is achievable in nearly half of this potentially treatment resistant group of patients. The mean time taken for 50% of the participants in each group to attain their first remission was 125 days for the CLZ group, and 135 days for the CLZ+CBT group. This increased to 180 days for the TDZ+CBT group, while 55% of the TDZ group remained unremitted at 220 days the last time at which data was recorded. This analysis was repeated including a correction for attendance at CBT sessions. For the 23 participants randomized to CBT, the mean number of sessions was 14.3 (SD = 8.1), while the median was 16, with a range of 0–24, and hence the CBT groups were further dichotomized into either attendance at <15 sessions, or ≥15 sessions. When attendance at CBT sessions was factored in to the analysis, the mean time taken by 50% of those in the CLZ+CBT group who had attended less than 15 sessions to reach their first remission was 128 days, decreasing to 102 days for those who had attended more than 15 sessions. For those in the TDZ+CBT group who attended more than 15 sessions, the time to 50% remission was 120 days, while only approximately 30% of those in the TDZ group who attended less than 15 sessions had attained their first remission by the end of the study period. 

## 4. Discussion

This investigation is among the few studies to use clozapine [24–26] within the six months of initial diagnosis in individuals with FEP (predominantly schizophrenia), whose positive symptoms remain prominent following initial treatment, and to offer a comparison and integration of psychological and pharmacological interventions in the early phase of illness. Due to the limitations associated with single-site recruitment and the highly restrictive nature of this patient group, only a small sample was able to be recruited. However, our cohort reflects the nature of this particular population in which factors such as first-time diagnosis, ambivalence about treatment, and the need for client collaboration during the engagement and treatment process often lead to high refusal rates and poor adherence to initial treatment in many. Despite the difficulties in interpretation that arise with small-scale pilot studies such as this, our observations raise several interesting points that merit further consideration. 

Firstly, our findings indicate that with appropriate treatment, symptomatic remission is attainable in a substantial percentage of FEP patients who are manifesting tenacious or persistent positive symptoms. As expected, the most rapid response was achieved with clozapine treatment, in line with several other studies involving individuals with FEP [24, 26]. Furthermore, a substantially greater percentage of the participants who received clozapine attained remission at some point during the study than in those who were treated with thioridazine (52% versus 35%). However, combining clozapine with CBT designed to target positive symptoms did not appear to confer a significant therapeutic advantage over treatment with medication alone since the both the time to response (125–135 days for 50% of the group to attain at least a first remission) and the percentage of participants remaining unremitted at the end of the study period (40%) were similar for both treatment groups. Thioridazine treatment was less effective than clozapine in terms of the response time and the remission rate, with over 55% of participants in this group remaining unremitted at the end of the study period. However, combining CBT with thioridazine treatment substantially reduced the response time (180 days for 50% of the group to attain at least one remission) and the percentage of participants remaining unremitted (40%). Analysis of these data after dichotomization of the groups receiving CBT into those who attended either less or more than half the CBT sessions offered accentuated these differences even further. Unsurprisingly, given the small numbers in each group, these analyses did not reach statistical significance, although the data suggests that CBT does benefit these patients by reducing the time taken to respond to treatment. 

Detailed examination of the psychopathology outcome measures supports this hypothesis. Treatment with clozapine resulted in a reduction in positive symptoms that was clearly evident at 12 weeks, while thioridazine gave a more modest response over this timeframe. Combining clozapine with CBT did not augment the treatment response, however, combining thioridazine with CBT gave a reduction in positive symptoms that was equivalent to that achieved with clozapine treatment alone, suggesting that augmenting slower-acting medications with CBT may provide an early therapeutic benefit as an alternative to instituting CLZ. Interestingly, while the improvements seen after treatment with either medication remained stable after the first 12 weeks of the study, augmenting either medication with CBT led to a further amelioration in positive symptoms over the final 12 weeks of the study. In terms of negative symptoms, treatment with either clozapine or thioridazine gave a relatively modest improvement, while combining CBT with either medication led to statistically significant improvements which were apparent by 12 weeks of treatment and were stably maintained until the end of the study period. Taken together, these observations suggest that CLZ and CBT may have an early beneficial effect on both positive and negative symptoms, which appears to be sustained for at least three months after the end of therapy. 

While only limited conclusions can be drawn from this study, the results indicate that clozapine treatment may be a useful therapeutic option for individuals with FEP who are experiencing persistent positive symptoms. The main advantages of clozapine treatment include a shorter response time and a higher rate of symptomatic remission than that achieved with thioridazine. Our findings are in agreement with those reported by Agid et al. [24] in a recent study of the efficacy of clozapine treatment in a small cohort of individuals with FEP who had not responded to treatment with two first-line atypical antipsychotics. These authors also observed a robust response to clozapine, with 77% of their cohort responding to treatment, where response was defined as a “much improved” or “very much improved” score on the CGI improvement scale, or a BPRS thought disorder subscale score of 6 or less. Apart from its more rapid effect on positive symptoms, we noted a further benefit of clozapine treatment that appears to be due to its intrinsic antidepressant activity: a significant early improvement in depressive symptoms in the clozapine group that did not occur in the other treatment groups. Meltzer has reported that the ability of clozapine to reduce psychopathology may be delayed until six to nine months in a significant proportion of patients [27], and thus future studies will benefit from extending the reporting time to 12 months to fully capture the delayed effects of this drug. 

Previous research has demonstrated that CBT may speed symptomatic remission from FEP [28, 29]. Here, we show that CBT appears to have a beneficial effect during the early phases of treatment, by reducing the intensity of both positive and negative symptoms and thus the time taken to respond to treatment, as well as a stabilizing effect over time. Indeed, it appears that the combination of CBT and thioridazine was equally as effective as clozapine alone in terms of symptomatic reduction, suggesting that CBT may well provide another tool for the management of persistent symptoms in FEP patients that have not responded to the more widely prescribed first-line atypical antipsychotics, particularly when combined with an alternative antipsychotic. CBT may also be offered as an alternative therapeutic option for certain low-risk first-episode clients who refuse antipsychotic treatment, when provided within the context of a specialized early psychosis service that offers intensive psychosocial interventions and support.

As this pilot ran over five years, it is difficult to see how a full version of this trial could be conducted at a single site. Moreover, the effect sizes of the various measures range from small to large. Using a medium effect size (0.25) as a guide, it would require a sample size of about 130. This highlights the need for large multicentre trials of treatment regimes for this client group. A further caveat associated with this study is that “treatment as usual” through the EPPIC service may be considered an assertive, highly supportive approach, including regular case management, group programs, employment and legal assistance, outreach through crisis assessment and treatment teams, and triage and a specialist inpatient unit. As this is a comprehensive program of support for clients and their families, it has proven difficult to observe additional benefits with specific interventions (e.g., [28, 30]). This implies that any advantages of a combination of clozapine and CBT may be more evident in less well-resourced clinical services, where reliance on treatment with medication alone is higher. Regardless, further large-scale multicentre trials are necessary to definitively establish the utility of these treatment modalities in treatment-resistant early psychosis patients.

## Figures and Tables

**Table 1:** Demographic and intervention data.

	TDZ (*n* = 11)	TDZ+CBT (*n* = 12)	CLZ (*n* = 14)	CLZ+C (*n* = 11)
Male (%)	72.7	58.3	64.3	90.9
Age in years, mean ± SD	22.5 ± 3.4	22.0 ± 4.1	20.5 ± 3.5	20.8 ± 2.8
Schizophrenia (%)	90.9	75.0	78.6	81.8
Schizophreniform (%)	9.1	16.7	14.3	18.2
Delusional disorder (%)	0	8.3	0	0
DUP in months, mean ± SD [median]	14.95 ± 15.9 [12.0]	15.55 ± 15.0 [12.3]	14.65 ± 14.2 [12.0]	13.75 ± 29.2 [2.7]
Max. CLZ dose (mg/day) 2 weeks pre-baseline, mean ± SD	340.15 ± 124.3	349.25 ± 151.7	326.55 ± 110.7	340.65 ± 108.9
Max. medication dose (mg/day) during trial, mean ± SD	316.75 ± 117.3	282.55 ± 163.3	298.55 ± 153.3	301.25 ± 195.8
Medication dose (mg/day) at 12 weeks, mean ± SD	268.65 ± 178.4	379.75 ± 345.2	315.15 ± 201.9	276.25 ± 211.6
Medication dose (mg/day) at 24 weeks, mean ± SD	148.55 ± 111.9	296.65 ± 274.8	364.65 ± 244.8	266.35 ± 182.6
No. of CBT sessions, mean ± SD, [median]		13.45 ± 9.6 [16.0]		15.25 ± 6.5 [19.0]
Outpatient hours at 12 weeks, mean ± SD	12.65 ± 13.2	6.75 ± 7.0	9.55 ± 9.2	8.55 ± 4.8
Inpatient hours + home interventions at 12 weeks	2.15 ± 6.9	14.25 ± 42.0	1.85 ± 4.6	14.85 ± 28.3

**Table 2:** Mean outcome measure scores at baseline and weeks 12 and 24 (as means and standard deviations). Missing values were provided using multiple imputation.

		*N*	Baseline	Week 12	Week 24
BPRS-P	TDZ	11	10.4 ± 1.9	9.3 ± 2.5	9.4 ± 2.5
	TDZ+CBT	12	11.2 ± 2.7	8.0 ± 1.3	6.5 ± 1.8
	CLZ	14	11.8 ± 2.2	8.4 ± 3.1	8.7 ± 2.5
	CLZ+CBT	11	12.2 ± 2.8	9.1 ± 4.2	7.9 ± 4.0

SANS	TDZ	11	32.9 ± 13.0	28.4 ± 8.6	23.5 ± 7.2
	TDZ+CBT	12	37.2 ± 9.3	23.9 ± 5.1*	25.9 ± 9.7
	CLZ	14	35.4 ± 11.8	29.4 ± 6.6	27.8 ± 8.0
	CLZ+CBT	11	40.2 ± 14.8	26.5 ± 12.1*	24.9 ± 12.2

BDI	TDZ	11	24.0 ± 6.5	22.9 ± 6.7	22.2 ± 6.7
	TDZ+CBT	12	29.0 ± 6.3	22.8 ± 4.4	17.0 ± 2.6*
	CLZ	14	26.6 ± 8.1	18.1 ± 5.5*	18.6 ± 4.9*
	CLZ+CBT	11	25.2 ± 7.8	22.5 ± 9.6	22.6 ± 7.6

CGI	TDZ	11	4.6 ± 0.5	3.6 ± 1.0	3.5 ± 0.8
	TDZ+CBT	12	4.8 ± 0.9	3.5 ± 0.8	3.0 ± 0.5
	CLZ	14	4.9 ± 0.7	3.6 ± 0.9	3.4 ± 0.8
	CLZ+CBT	11	4.8 ± 0.9	3.6 ± 1.4	3.5 ± 0.8

SOFAS	TDZ	11	47.8 ± 12.6	53.2 ± 13.8	55.2 ± 12.3
	TDZ+CBT	12	43.0 ± 12.6	50.4 ± 9.2	55.4 ± 5.8
	CLZ	14	44.8 ± 9.9	54.0 ± 9.6	53.8 ± 9.8
	CLZ+CBT	11	45.2 ± 10.6	52.1 ± 11.1	55.4 ± 10.5

QLS	TDZ	11	48.7 ± 20.0	53.0 ± 17.2	59.6 ± 13.7
	TDZ+CBT	12	44.3 ± 14.2	49.8 ± 11.4	47.1 ± 13.1
	CLZ	14	50.2 ± 12.9	58.8 ± 10.4	54.5 ± 14.6
	CLZ+CBT	11	54.8 ± 15.7	59.3 ± 11.9	63.3 ± 16.1

*Statistically significant, *P* ≤ .05.
